# Estimating causal associations of atopic dermatitis with depression using the propensity score method: an analysis of Korea Community Health Survey data, 2010-2013

**DOI:** 10.4178/epih.e2018059

**Published:** 2018-11-29

**Authors:** Hayon Michelle Choi, Dahye Kim, Whanhee Lee, Ho Kim

**Affiliations:** Graduate School of Public Health, Seoul National University, Seoul, Korea

**Keywords:** Atopic dermatitis, Depression, Epidemiology, Propensity score

## Abstract

**OBJECTIVES:**

Numerous studies have reported associations between atopic dermatitis (AD) and depression, but the causal relationship between the 2 diseases has not been established. Therefore, this study used the propensity score method to investigate whether there was a positive causal effect of AD on depression in 16 regions (cities and provinces) in Korea.

**METHODS:**

The study analyzed 16 regions (cities and provinces) in Korea, using data obtained from the Korea Community Health Survey for the years 2010-2013. Propensity score matching was used to estimate the causal influence of AD on depression in Korea.

**RESULTS:**

After propensity score matching, the standardized difference for each covariate among the 16 regions (cities and provinces) was less than 1, indicating a balance between the case and control groups. At the national level, those diagnosed with AD had a 2.31 times higher risk for being diagnosed with depression than those who had not been diagnosed with AD. In particular, the risk was highest in North Jeolla Province (odds ratio [OR], 4.87; 95% confidence interval [CI], 2.28 to 10.43) and lowest in Gwangju (OR, 1.82; 95% CI, 0.87 to 3.79), and the OR for Seoul was 2.23 (95% CI, 1.66 to 2.99).

**CONCLUSIONS:**

This study provides insights into how causal inferences can be derived from observational studies, through an analysis of Korea Community Health Survey data. Furthermore, the study results have implications for region-specific guidelines for preventive health policies targeting depression.

## INTRODUCTION

Atopic dermatitis (AD) is an inflammation of the skin accompanied by itchiness, and numerous studies conducted throughout the world have reported AD to be associated with depression [1-5]. A study conducted in Taiwan among adolescents and adults with AD found a positive association between AD and depression in both groups, although the risk of AD for depression was higher among adults than among adolescents [1]. Furthermore, a study of 257 AD patients in the US found a significant association between the severity of AD and depression [6]. Studies reporting an association between AD and depression in Korea have also been published [7-9]. A study using Korea Community Health Survey (KCHS) data from 2008 to 2013 presented a 1.33 times (95% confidence interval [CI], 1.02 to 1.74) higher risk of depression in AD patients than in non-AD patients [9]. Furthermore, adolescents with AD were 1.27 times more likely to experience depression symptoms [7]. The prevalence of both depression and AD is increasing in Korea [10,11]; therefore estimating the risk of AD for depression is required, along with an analysis of suitable medical and health policies.

Most observational investigations have shown statistically significant associations between AD and depression, although few studies have sought to characterize the causal relationship between these 2 diseases [12]. The symptoms of AD (itchiness, aches, etc.) cause a lack of sleep and difficulties concentrating [13], leading to stress in social relationships [14,15]. In particular, numerous cytokines and immune cells are involved in the pathogenesis of AD and might interact with factors influencing sleep [16]. These results of biological and social studies support the causal effect of AD on depression. However, in observational studies, selection bias occurs and the distribution of covariates among the exposed and unexposed groups is not homogeneous since the measured variables cannot be controlled. The heterogeneity of covariates between 2 groups limits the ability to draw causal inferences [17-20]. To overcome these limitations of observational studies, randomized clinical trials (RCTs) and cohort studies are conducted to estimate causal associations. Nevertheless, such designs are not suitable for large-scale epidemiological studies due to their high costs and difficulties with sampling [21,22].

Several methods have been proposed to overcome selection bias in observational studies [19,23,24]. Among those methods, the most commonly used is propensity score matching (PSM) [18]. The propensity score is the conditional probability that an individual will belong to the exposed group when a specific covariate value is given [18]. In PSM, study subjects who are matched by the levels of covariates affecting the results are assigned to exposed and unexposed groups. This method is similar to an RCT, in that randomly assigning differences in covariance between the exposed and unexposed groups minimizes selection bias in observational studies [17]. Furthermore, PSM can be conducted not only in observational studies, but also in retrospective studies, for which random assignment is difficult [25]. Thus, our study aimed to estimate the causal association between AD and depression diagnoses using the PSM approach.

## MATERIALS AND METHODS

### Data

We analyzed data from the KCHS during 2010-2013 for 16 regions (cities and provinces ) in Korea [20]. The KCHS was organized by the Korea Centers for Disease Control and Prevention [26]. Data were collected from adults more than 19 years old through interviews. First, the samples for the KCHS were selected from an average of 900 adults per region (city, county, district) based on the housing type for each township, neighborhood, and town. Probability proportional to size systematic sampling was used for the first sample region, and then the sample families were selected as the secondary sample region [27]. A total of 917,948 people were included in the KCHS data used in this study (2010: 230,712; 2011: 229,229; 2012: 229,226; 2013: 228,781). Using the data, we calculated participants’ body mass index (BMI), and used self-reported data for level of education, current smoking status, current drinking status, preference for low sodium intake, diagnosis of AD, and diagnosis of depression.

### Statistical analysis

PSM was used to estimate the causal association between AD and depression [28]. The outcome was the self-reported diagnosis of depression by a doctor at any point in the respondent’s lifetime. The subjects were asked “Have you ever been diagnosed with depression by a doctor?”. The exposure variable was a self-reported AD diagnosis, which was obtained by asking the subjects “Have you ever been diagnosed with AD by a doctor?”. Eight other covariates were considered (age, sex, current smoking status, current drinking status, preference for low sodium intake, BMI, and year). To take into account the time trend of depression diagnoses, an indicator variable for time was added [29]. The study utilized a 3-stage analysis.

#### First stage: propensity score matching

For the composite sample design of the KCHS, a logistic propensity model considering weighting, stratification, and cluster variables is shown in equation (1).

(1) logPZ=1χ1,χ2,...χpPZ=0χ1,χ2,...χp=β0+∑t=1pβtχt

AD diagnosis is indicated as Z (Z=1 when diagnosed with AD [case], Z=0 when not diagnosed with AD [control]), and *x_t_* indicates covariates. Using the following equation, the propensity score was calculated. The propensity score could be expressed as equation (2).

(2) P^Z=1χ1,χ2,...,χp=expβ^0+∑t=1pβ^tχt1+expβ^0+∑t=1pβ^tχt=propensity score

Based on the propensity score from equation (2), each case was matched at a 1:1 ratio 19 with the closest propensity score. Specifically, the caliper method matches each case 20 within a certain logit propensity score $\left(\log\frac{\left(\mathrm{propensity}\;\mathrm{score}\right)}{1-\left(\mathrm{propensity}\;\mathrm{score}\right)}\right)$ range, which is generally defined as 0.2 times the standard deviation of the propensity score [30,31]. In this study, a 0.28 caliper range was used in the analysis. The first-stage analysis was conducted using SAS version 9.4 (SAS Institute Inc., Cary, NC, USA).

#### Second stage: conditional logistic regression

(3) $$\mathrm{clogit}(P_{i\;})=\tau {\;}_{0\;i}\;+\gamma_{\;i}\;Z{\;}_{i\;},\;i=\;1,\;2,\;3,\;\ldots 15,\;16$$

In the second stage, a conditional logistic regression model was fitted to each region using the 1:1 matched data from the first stage. The conditional logistic regression analysis was conducted using the Breslow method. *γ _i_* is the region with the ith coefficient of the AD diagnosis (Z i), and *P _i_* is the expected probability that the subject will have depression in region *i*.

#### Third stage: meta-analysis

(4) $$W{\;}_{i}=\;\mu \;+\;\xi {\;}_{i}+\;\varepsilon {\;}_{i\;},\;\;\;i=\;1,\;2,\;3,\;\ldots 15,\;16$$

A random-effect multivariate meta-analysis was computed, as shown in equation (4). The observed effect (*W_i_*) was expressed as the true variation in the effect size (*ξ _i_*), summary effect (*μ*), and the sampling error (*ε _i_*). *ξ _i_* is the distance between the effect for each 16 coefficients of the AD diagnosis (*γ _i_*). Furthermore, the region population was considered as the weight in the third stage. The causal association of AD on depression diagnosis was expressed in terms of ORs. The second and third stages of the analysis were performed using R version 3.4.2 (https://cran.r-project.org/bin/windows/base/old/3.4.2/) and the R package *metaphor* [32]. For a sensitivity analysis, we applied different propensity methods, including stratification, propensity score adjustment as a covariate, and weighting. In stratification, subjects are grouped into mutually exclusive subsets by the propensity score, resulting in a similar propensity score within each stratum between the exposed and unexposed subjects. A widely used approach in stratification is to divide subjects into 5 equal-size quintiles [33]. In covariate adjustment, the developed propensity score is assumed as a predictor variable . This separate multivariable regression model estimates the outcome, while adjusting for the probability that a subject belongs to the exposed group. For weighting, the inverse probability of treatment weighting using the propensity score was employed. This concept corresponds to survey sampling weights, which make a sample representative of a specific population [34].

## RESULTS

Table 1 presents the overall descriptive statistics according to AD diagnosis before and after PSM. Before PSM, there were 895,046 subjects not diagnosed with AD and 21,111 diagnosed with AD . After PSM, there were 21,111 subjects in both groups. The distribution of covariates between cases (AD diagnosis) and controls (no AD diagnosis) after PSM was similar. Of the controls, 65.3% were current drinkers before PSM, and after PSM the proportion was 26.7%, which was identical to the proportion of current drinkers among cases (26.7%). Furthermore, the standardized difference in each covariate sharply decreased after PSM, from 2.19-54.56 to 0.01-0.22. The p-value of the chi-square test for the standardized difference is shown below in Table 1. The findings accepted the null hypothesis that there would be no differences between cases and controls after PSM (p=0.42) (H_0_: the difference between the two groups is zero). Supplementary Material 1 presents descriptive statistics for each of the 16 cities and provinces.

**Figure 1.**
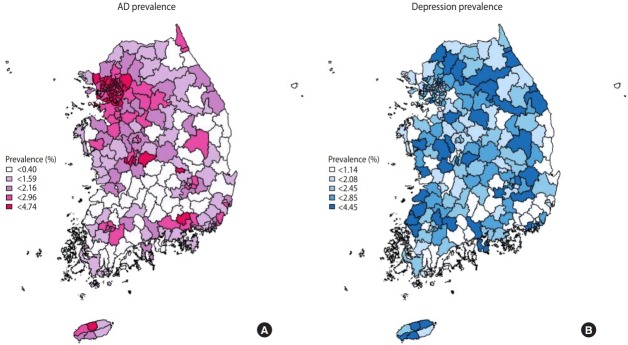
Geographical distribution of the prevalence of (A) atopic dermatitis (AD) and (B) depression diagnoses.

Figure 1 is a national map representing the standardized prevalence of AD and depression diagnoses considering the sampling weight. AD diagnoses in all administrative subunits of Seoul showed the highest prevalence (2.72-4.74%), while the lowest prevalence was found in South Jeolla Province (0.47-2.73%). The prevalence of AD diagnoses tended to be higher in metropolitan areas (Seoul, Gyeonggi Province). The prevalence of depression diagnoses was highest in Jeju Province (2.15-3.21%) and lowest in Ulsan (1.23-2.27%). The prevalence of depression diagnoses showed wide geographical variation across the country. The prevalence of AD and depression diagnoses in the 253 administrative divisions of Korea ar all administrative subunits level is demonstrated in Supplementary Material 2.

**Figure 2.**
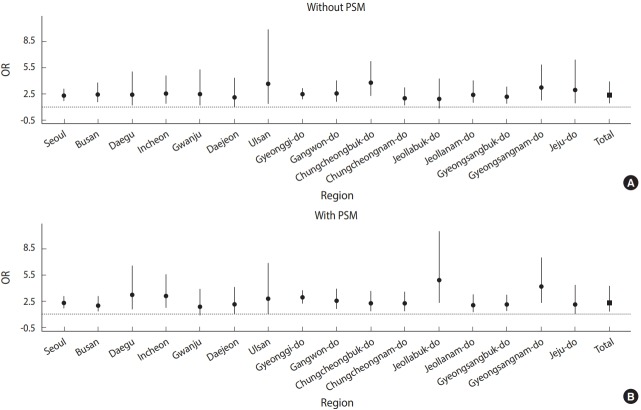
Odds ratios (ORs) from the meta-analysis (A) without propensity score matching (PSM) and (B) with PSM.

The risk of receiving a depression diagnosis among those with an AD diagnosis in the 16 cities and provinces before and after PSM is presented as ORs in Table 2 and Figure 2. After PSM, people in Korea with an AD diagnosis were 2.31 times (95% CI, 1.92 to 2.76) more likely to have been diagnosed with depression. The risk was highest in North Jeolla Province (OR, 4.87; 95% CI, 2.28 to 10.43) and lowest in Gwangju (OR, 1.82; 95% CI, 0.87 to 3.79). The risk also differed by region based on the use of PSM; people diagnosed with AD were 2.23 times (95% CI, 1.66 to 2.99) more likely to have received a depression diagnosis when using PSM, while the risk was 2.26 (95% CI, 1.67 to 3.05) before PSM.

The ORs when using stratification, covariate adjustment, and the propensity score weighting method are shown in Table 3. When stratification was used, the propensity score within the first to fifth quartiles had ORs with a range of 1.88-2.47. The OR was 2.36 (95% CI, 1.48 to 3.74) using covariate adjustment, and 2.38 (95% CI, 1.28 to 4.42) using the weighting method. These findings were similar to the OR of 2.38 obtained using PSM.

## DISCUSSION

This study estimated the causal effect of AD on depression diagnosis using data from the KCHS. Before PSM, each covariate ratio was unbalanced, but the covariates became balanced after PSM (overall p-value=0.42). In this analysis using PSM to conduct an analysis of 16 regions in Korea, it was found that individuals diagnosed with AD had a 2.31 times (95% CI, 1.92 to 2.76) higher risk of having been diagnosed with depression than their counterparts.

Several studies have documented biological mechanisms through which AD may influence the likelihood of a depression diagnosis [35-38]. Depression is associated with changes in the immune system [39-42], and immunoglobulin E (IgE)–mediated allergies are more common in depression patients than other allergies [35]. Since AD is an IgE-mediated disease [43], AD patients are likely to have more severe depression symptoms [37,38]. Thus, immune mediators, such as cytokines, are involved in the mechanism underlying both AD and depression [44-47]. Cytokines mediate the chemical communications between the immune system and the brain [48]. In AD, the cytokine interleukin-4 affects serotonin (5-HT) metabolism [47]. Specifically, 5-HT is an essential mediator of both the nervous and the immune systems, and some aspects of the role of 5-HT in the body have been linked to depression [47]. Therefore, depression could be explained by changes in 5-HT metabolism during AD [49].

Studies have reported that AD is closely related to depression, and may actually be an underlying cause of depression. Considering demographic, socioeconomic, and clinical characteristics, 1:5- matched PSM was conducted using KCHS data from 2007-2012 [8]. The results showed a 1.36 times higher risk of depression in AD patients. Another study in Korea among adolescents demonstrated that AD was associated with an OR for depression of 1.28 (95% CI, 1.20 to 1.37), an OR of suicidal thoughts of 1.31 (95% CI, 1.21 to 1.42), an OR of suicidal planning of 1.42 (95% CI, 1.26 to 1.61), and an OR for suicide attempt of 1.51 (95% CI, 1.32 to 1.73) [7].

This study has some strengths. First, it estimated the causal association between these 2 variables by adjusting for the confounding factors in an observational study setting. When attempting to characterize a causal relationship, it is difficult to arrive at an estimate using methods other than an RCT. However, RCTs have limitations in terms of time, ethical issues, and costs; thus, many studies are based on observational data, for which PSM could be a practical method, such as in this study. Second, this study considered the local characteristics of the 16 regions. When calculating the propensity score, the stratification variables, cluster variables, and weights were considered in order to reflect the KCHS questionnaire design. We also reduced discrepancies between regions by weighting each population while deriving the overall risk. Third, this study presented findings on the causal association between AD and depression in Korea. Even though studies have shown associations between AD and depression, few studies have estimated the causal effect of AD on depression in Korea. Furthermore, this study presented several results obtained using propensity score methods for 16 regions.

There are some inherent limitations of the propensity score method. When calculating the propensity score, all covariates used in the analysis should not have missing values. Since considering all covariates is problematic, the ability to characterize the causal relationship perfectly is limited. Furthermore, the propensity score method is a statistical method that cannot alter the fundamental research design. Moreover, since there could be an association between spatial clustering and the disease incidence, further studies should be conducted using spatial clustering analysis.

This study proposed a causal effect of AD on depression diagnosis using data from the KCHS, and suggested ideas and methods for estimating causal associations based on an observational study. Our study results make an implication for preventive health interventions targeted toward AD patients at risk for depression and geographical areas vulnerable to AD.

## Figures and Tables

**Table 1.** Descriptive statistics and standardized differences for individuals with and without AD, before and after PSM

Variables		No AD		AD (n=21,111)	Standardized difference	
		Before PSM (n=895,046)	After PSM (n=21,111)		Before PSM	After PSM
Age (yr)		51.4±16.8	42.2±17.9	41.8±17.8	54.56	0.22
Sex	Male	404,596 (45.2)	8,634 (40.9)	8,616 (40.8)	9.79	0.14
	Female	490,450 (54.8)	12,477 (59.9)	12,495 (59.2)	-	-
BMI (kg/m^2^)		22.9±3.4	22.4±3.1	22.6±3.4	6.87	0.01
Education level	No education	73,450 (8.2)	902 (4.3)	802 (3.8)	41.13	0.27
	Preschool	2,483 (0.3)	17 (0.1)	34 (0.2)	-	-
	Elementary school	164,484 (18.3)	2,342 (11.1)	2,301 (10.9)	-	-
	Middle school	104,443 (11.7)	1,598 (7.6)	1,603 (7.6)	-	-
	High school	260,909 (29.1)	5,286 (25.0)	5,364 (25.4)	-	-
	2-3 years of college	91,958 (10.3)	3,319 (16.1)	3,391 (16.1)	-	-
	4 years of university	169,648 (18.9)	6,884 (32.6)	6,688 (31.7)	-	-
Current drinking	Yes	584,163 (65.3)	5,557 (26.7)	5,640 (26.7)	16.43	0.02
	No	310,883 (34.7)	15,554 (73.3)	15,471 (73.3)	-	-
Current smoking	Yes	175,772 (19.6)	3,643 (17.3)	3,999 (18.9)	2.19	0.15
	No	719,274 (80.4)	17,469 (82.7)	17,112 (8.1)	-	-
Sodium intake	Very high	8,119 (0.9)	247 (1.2)	318 (1.5)	7.38	0.16
	High	228,121 (25.5)	6,411 (30.4)	6,424 (30.4)	-	-
	Moderate	453,192 (50.6)	9,678 (45.8)	9,405 (44.6)	-	-
	Low	185,801 (20.8)	4,409 (20.9)	4,437 (21.0)	-	-
	Very low	19,634 (2.2)	364 (1.7)	521 (2.5)	-	-
Depression	Yes	21,788 (2.4)	427 (2.0)	1,063 (5.0)	-	-
	No	873,258 (97.6)	20,684 (98.0)	20,048 (95.0)	-	-
p-value^1^					<0.001	0.42

Values are presented as mean±standard deviation or number (%). AD, atopic dermatitis; PSM, propensity score matching.

1 Using the chi-square test for the standardized difference of covariate means.

**Table 2.** ORs before and after PSM

Region	Before PSM	After PSM
Seoul	2.26 (1.67, 3.05)	2.23 (1.66, 2.99)
Busan	2.39 (1.54, 3.72)	1.94 (1.26, 2.97)
Daegu	2.37 (1.14, 4.96)	3.20 (1.57, 6.51)
Incheon	2.50 (1.37, 4.54)	3.07 (1.71, 5.49)
Gwangju	2.45 (1.14, 5.27)	1.82 (0.87, 3.79)
Daejeon	2.07 (0.99, 4.30)	2.08 (1.07, 4.03)
Ulsan	3.67 (1.38, 9.80)	2.67 (1.04, 6.81)
Gyeonggi Province	2.42 (1.88, 3.11)	2.88 (2.26, 3.69)
Gangwon Province	2.54 (1.61, 4.01)	2.50 (1.61, 3.88)
North Chungcheong Province	3.76 (2.29, 6.19)	2.21 (1.36, 3.58)
South Chungcheong Province	1.95 (1.19, 3.20)	2.17 (1.34, 3.51)
North Jeolla Province	1.89 (0.86, 4.16)	4.87 (2.28, 10.43)
South Jeolla Province	2.41 (1.46, 4.00)	2.00 (1.23, 3.26)
North Gyeongsang Province	2.11 (1.37, 3.23)	2.09 (1.38, 3.17)
South Gyeongsang Province	3.20 (1.76, 5.80)	4.14 (2.31, 7.43)
Jeju Province	2.97 (1.39, 6.35)	2.09 (1.02, 4.29)
Total	2.35 (1.44, 3.83)	2.31 (1.92, 2.76)

Values are presented as OR (95% confidence interval). OR, odds ratio; PSM, propensity score matching.

**Table 3.** Odds ratios after propensity score stratification, covariate adjustment, and weighting

Region	Stratification (quantile)					Covariate adjustment	Weighting
	First	Second	Third	Fourth	Fifth		
Seoul	2.06 (1.32, 3.23)	2.20 (1.44, 3.36)	1.82 (1.08, 3.06)	2.26 (1.52, 3.38)	2.12 (1.56, 2.88)	2.13 (1.78, 2.54)	2.14 (1.55, 2.96)
Busan	2.06 (1.06, 3.97)	1.18 (0.51, 2.70)	2.67 (1.40, 5.09)	3.91 (2.32, 6.59)	2.42 (1.42, 4.13)	2.49 (1.90, 3.25)	2.50 (1.60, 3.91)
Daegu	1.96 (0.78, 4.97)	2.89 (1.34, 6.22)	2.73 (1.14, 6.54)	1.73 (0.62, 4.83)	2.14 (1.04, 4.41)	2.24 (1.55, 3.25)	2.10 (1.15, 3.83)
Incheon	1.89 (0.68, 5.30)	3.09 (1.45, 6.57)	3.22 (1.51, 6.84)	2.62 (1.18, 5.80)	3.50 (2.07, 5.92)	3.01 (2.19, 4.13)	3.10 (1.75, 5.48)
Gwanju	1.84 (0.42, 8.05)	0.61 (0.08, 4.62)	5.22 (1.93, 14.1)	3.53 (0.95, 13.07)	3.17 (1.46, 6.87)	2.68 (1.66, 4.32)	2.63 (1.15, 6.04)
Daejeon	5.67 (2.30, 13.99)	1.56 (0.47, 5.13)	2.28 (0.67, 7.81)	1.25 (0.38, 4.15)	3.19 (1.61, 6.33)	2.41 (1.59, 3.65)	2.57 (1.21, 5.47)
Ulsan	1.34 (0.17, 10.34)	3.45 (1.00, 11.94)	4.79 (1.55, 14.83)	3.37 (0.93, 12.3)	3.53 (1.32, 9.47)	3.27 (1.91, 5.57)	3.90 (1.47, 10.37)
Gyeonggi Province	2.44 (1 .75, 3.40)	2.32 (1.66, 3.25)	2.66 (1.89, 3.73)	2.28 (1.64, 3.19)	2.43 (1.91, 3.10)	2.45 (2.14, 2.81)	2.46 (1.92, 3.14)
Gangwon Province	1.31 (0.53, 3.24)	3.20 (1.80, 5.68)	3.46 (1.99, 6.02)	2.75 (1.55, 4.88)	2.39 (1.50, 3.82)	2.65 (2.05, 3.41)	2.69 (1.75, 4.15)
North Chungcheong Province	5.06 (2.47, 10.35)	1.79 (0.77, 4.17)	3.56 (1.90, 6.69)	1.60 (0.73, 3.50)	3.18 (1.91, 5.31)	2.77 (2.06, 3.70)	2.77 (1.68, 4.57)
South Chungcheong Province	3.71 (2.05, 6.70)	0.96 (0.39, 2.37)	2.01 (1.04, 3.89)	1.33 (0.64, 2.76)	2.17 (1.25, 3.77)	1.94 (1.45, 2.60)	1.98 (1.23, 3.19)
North Jeolla Province	2.08 (0.75, 5.77)	4.38 (2.05, 9.37)	3.25 (1.53, 6.88)	2.44 (1.15, 5.18)	2.14 (1.04, 4.37)	2.78 (1.97, 3.92)	2.74 (1.57, 4.80)
South Jeolla Province	0.72 (0.18, 2.96)	2.40 (1.16, 4.98)	1.96 (0.95, 4.04)	3.51 (1.95, 6.33)	2.91 (1.75, 5.09)	2.49 (1.84, 3.36)	2.47 (1.53, 3.98)
North Gyeongsang Province	2.98 (1.55, 5.76)	3.38 (1.88, 6.08)	2.30 (1.29, 4.11)	2.48 (1.47, 4.19)	2.01 (1.21, 3.34)	2.49 (1.94, 3.21)	2.38 (1.60, 3.55)
South Gyeongsang Province	2.52 (1.16, 5.50)	2.06 (0.99, 4.29)	3.65 (1.93, 6.93)	3.03 (1.61, 5.70)	4.05 (2.51, 6.55)	3.11 (2.36, 4.10)	3.21 (2.01, 5.11)
Jeju Province	2.34 (0.81, 6.75)	0.86 (0.21, 3.60)	1.69 (0.51, 5.60)	1.84 (0.43, 7.96)	3.11 (1.52, 6.37)	2.03 (1.29, 3.22)	2.20 (1.02, 4.74)
Total	2.19 (1.03, 4.65)	1.88 (0.87, 4.07)	2.41 (1.13, 5.17)	2.40 (1.17, 4.93)	2.47 (1.34, 4.55)	2.36 (1.48, 3.74)	2.38 (1.28, 4.42)

Values are presented as odds ratio (95% confidence interval).

## SUPPLEMENTARY MATERIALS

Supplementary Material 1: Table S1 is available at http://www.e-epih.org/).

Supplementary Material 2: Table S2 is available at http://www.e-epih.org/).
