# Supplementary material for: Estimating causal associations of atopic dermatitis with depression using the propensity score method: an analysis of Korea Community Health Survey data, 2010-2013
Source: Epidemiol Health. 2018 Nov 29;40:e2018059. doi: 10.4178/epih.e2018059 (PMC6335500; doi:10.4178/epih.e2018059)
Supplement: Supplementary file 1 [file epih-40-e2018059-supplementary1.pdf]

Supplementary Material 1

Table S1. Descriptive statistics and standardized difference for not AD and AD: before and after PSM in 253 study communities in South Korea

Regions	Variables		Not AD		AD
			Before PSM	After PSM	
Seoul	Total		88,783	3,447	3,447
	Age (yrs, sd)		47 (16.1)	38.7 (16.4)	38.1 (16.2)
	Sex (n, %)	Male	39,268 (44.20%)	1,434 (41.60%)	1,440 (41.80%)
		Female	49,515 (55.80%)	2,013 (58.40%)	2,007 (58.20%)
	BMI (kg/m^2, sd)		2,270 (2.70%)	77 (2.40%)	65 (2.10%)
	Education level (n, %)	No education	99 (0.10%)	2 (0.10%)	0 (0.00%)
		Pre-school	8,421 (10.20%)	173 (5.40%)	174 (5.50%)
		Elementary school	8,278 (10.00%)	203 (6.40%)	184 (5.80%)
		Middle school	24,868 (30.00%)	689 (21.70%)	714 (22.60%)
		High school	9,776 (11.80%)	543 (17.10%)	563 (17.80%)
		2-3yrs college	29,060 (35.10%)	1,495 (47.00%)	1,460 (46.20%)
		4yrs university	65,336 (73.60%)	2,713 (78.70%)	2,737 (79.40%)
	Current drinking (n, %)	Yes	23,447 (26.40%)	734 (21.30%)	710 (20.60%)
		No	16,098 (18.10%)	590 (17.10%)	642 (18.60%)
	Current smoking (n, %)	Yes	72,685 (81.90%)	2,857 (82.90%)	2,805 (81.40%)
		No	860 (1.00%)	36 (1.00%)	45 (1.30%)
	Sodium intake (n, %)	Very high	22,411 (25.20%)	1,095 (31.80%)	1,098 (31.90%)
		High	43,602 (49.10%)	1,495 (43.40%)	1,439 (41.70%)
		Moderate	19,294 (21.70%)	733 (21.30%)	754 (21.90%)
		Low	2,598 (2.90%)	88 (2.60%)	111 (3.20%)
		Very low	2,255 (2.50%)	69 (2.00%)	160 (4.60%)
	Depression (n, %)	Yes	86,528 (97.50%)	3,378 (98.00%)	3,287 (95.40%)
		No	86,528 (97.5%)	3,378 (98.0%)	3,287 (95.4%)
Busan	Total		57,244	1,287	1,287
	Age (yrs, sd)		49.5 (16.2)	41.8 (18)	41.5 (17.9)
	Sex (n, %)	Male	25,621 (44.80%)	514 (39.90%)	526 (40.90%)
		Female	31,623	773	761

			(55.20%)	(60.10%)	(59.10%)
	BMI (kg/m[2], sd)		23 (3.1)	22.4 (2.9)	22.7 (3.3)
	Education level (n, %)	No education	2,180 (3.90%)	42 (3.30%)	38 (3.10%)
		Pre-school	76 (0.10%)	0 (0.00%)	2 (0.20%)
		Elementary school	7,897 (14.20%)	109 (8.70%)	108 (8.70%)
		Middle school	7,753 (14.00%)	108 (3.40%)	107 (8.60%)
		High school	17,774 (32.00%)	315 (25.10%)	316 (25.40%)
		2-3yrs college	6,368 (11.50%)	203 (16.20%)	211 (17.00%)
		4yrs university	13,505 (24.30%)	478 (38.10%)	462 (37.10%)
	Current drinking (n, %)	Yes	41,337 (72.20%)	999 (77.60%)	1005 (78.10%)
		No	15,907 (27.80%)	288 (22.40%)	282 (21.90%)
	Current smoking (n, %)	Yes	11,532 (20.10%)	229 (17.80%)	260 (20.20%)
		No	45,712 (79.90%)	1,058 (82.20%)	1,027 (79.80%)
	Sodium intake (n, %)	Very high	477 (0.80%)	15 (1.20%)	26 (2.00%)
		High	15,270 (26.70%)	400 (31.10%)	416 (32.30%)
		Moderate	27,824 (48.60%)	559 (43.40%)	530 (41.20%)
		Low	12,311 (21.50%)	292 (22.70%)	281 (21.80%)
		Very low	1,361 (2.40%)	21 (1.60%)	34 (2.60%)
	Depression (n, %)	Yes	1,450 (2.50%)	33 (2.60%)	62 (4.80%)
		No	55,794 (97.50%)	1,254 (97.40%)	1,225 (95.20%)
Daegu	Total		28,608	704	704
	Age (yrs, sd)		48.5 (16.2)	41.2 (17.6)	40.8 (17.9)
	Sex (n, %)	Male	12,984 (36.90%)	289 (41.10%)	316 (44.90%)
		Female	15,624 (44.40%)	415 (58.90%)	388 (55.10%)
	BMI (kg/m[2], sd)		22.8 (3.1)	22.4 (2.9)	22.6 (3.1)
	Education level (n, %)	No education	1,117 (4.10%)	21 (3.10%)	14 (2.10%)
		Pre-school	44 (0.20%)	52 (7.70%)	61 (9.10%)
		Elementary school	4,152 (15.10%)	0 (0.00%)	0 (0.00%)
		Middle school	3,345 (12.10%)	51 (1.60%)	53 (7.90%)
		High school	8,192 (29.70%)	163 (24.20%)	169 (25.20%)
		2-3yrs college	4,040 (14.70%)	118 (17.50%)	130 (19.40%)
		4yrs university	6,675 (24.20%)	268 (39.80%)	244 (36.40%)

	Current drinking (n, %)	Yes	20,202 (70.60%)	545 (77.40%)	528 (75.00%)
		No	8,406 (29.40%)	159 (22.60%)	176 (25.00%)
	Current smoking (n, %)	Yes	6,004 (21.00%)	122 (17.30%)	127 (18.00%)
		No	22,604 (79.00%)	582 (82.70%)	577 (82.00%)
	Sodium intake (n, %)	Very high	256 (0.90%)	8 (1.10%)	9 (1.30%)
		High	8,176 (28.60%)	242 (34.40%)	240 (34.10%)
		Moderate	13,145 (46.00%)	295 (41.90%)	284 (40.30%)
		Low	6,290 (22.00%)	149 (21.20%)	154 (21.90%)
		Very low	738 (2.60%)	10 (1.40%)	17 (2.40%)
	Depression (n, %)	Yes	810 (2.30%)	10 (1.40%)	35 (5.00%)
		No	34,390 (97.70%)	694 (98.60%)	669 (95.00%)
Incheon	Total		35,200	926	926
	Age (yrs, sd)		49 (16.5)	39.2 (16.5)	39 (16.2)
	Sex (n, %)	Male	15,765 (55.10%)	390 (42.10%)	377 (40.70%)
		Female	19,435 (67.90%)	536 (57.90%)	549 (59.30%)
	BMI (kg/m^2, sd)		23 (3.4)	22.5 (3.6)	22.5 (3.6)
	Education level (n, %)	No education	1,632 (4.80%)	20 (2.20%)	14 (1.60%)
		Pre-school	59 (0.20%)	0 (0.00%)	0 (0.00%)
		Elementary school	5,006 (14.60%)	73 (8.10%)	65 (7.30%)
		Middle school	4,150 (12.10%)	58 (1.80%)	61 (6.80%)
		High school	12,935 (37.70%)	289 (32.10%)	305 (34.20%)
		2-3yrs college	4,109 (12.00%)	171 (19.00%)	171 (19.10%)
		4yrs university	6,389 (18.60%)	290 (32.20%)	277 (31.00%)
	Current drinking (n, %)	Yes	23,639 (67.20%)	734 (79.30%)	713 (77.00%)
		No	11,561 (32.80%)	192 (20.70%)	213 (23.00%)
	Current smoking (n, %)	Yes	7,317 (20.80%)	176 (19.00%)	195 (21.10%)
		No	27,883 (79.20%)	750 (81.00%)	731 (78.90%)
	Sodium intake (n, %)	Very high	350 (1.00%)	9 (1.00%)	13 (1.40%)
		High	8,880 (25.20%)	272 (29.40%)	273 (29.50%)
		Moderate	17,910 (50.90%)	448 (48.40%)	424 (45.80%)
		Low	7,281 (20.70%)	181 (19.50%)	196 (21.20%)
		Very low	770 (2.20%)	16 (1.70%)	20 (2.20%)

	Depression (n, %)	Yes	789 (2.80%)	16 (1.70%)	50 (5.40%)
		No	27,819 (97.20%)	910 (98.30%)	876 (94.60%)
Gwanju	Total		18,078	495	495
	Age (yrs, sd)		47.7 (16.2)	37.9 (16.8)	36.5 (15.9)
	Sex (n, %)	Male	8,256 (45.70%)	207 (41.80%)	204 (41.20%)
		Female	9,822 (54.30%)	288 (58.20%)	291 (58.80%)
	BMI (kg/m^2, sd)		22.8 (3.1)	22.3 (3)	22.3 (3.3)
	Education level (n, %)	No education	669 (3.90%)	13 (2.70%)	11 (2.30%)
		Pre-school	38 (0.20%)	1 (0.20%)	1 (0.20%)
		Elementary school	2,150 (12.50%)	36 (7.50%)	24 (5.10%)
		Middle school	1,833 (10.60%)	15 (0.50%)	24 (5.10%)
		High school	4,960 (28.80%)	99 (20.60%)	107 (22.60%)
		2-3yrs college	2,373 (13.80%)	82 (17.00%)	85 (18.00%)
		4yrs university	5,213 (30.20%)	235 (48.90%)	221 (46.70%)
	Current drinking (n, %)	Yes	12,961 (71.70%)	394 (79.60%)	386 (78.00%)
		No	5,117 (28.30%)	101 (20.40%)	109 (22.00%)
	Current smoking (n, %)	Yes	3,285 (18.20%)	85 (17.20%)	84 (17.00%)
		No	14,793 (81.80%)	410 (82.80%)	411 (83.00%)
	Sodium intake (n, %)	Very high	152 (0.80%)	12 (2.40%)	9 (1.80%)
		High	4,762 (26.30%)	158 (31.90%)	164 (33.10%)
		Moderate	9,414 (52.10%)	234 (47.30%)	225 (45.50%)
		Low	3,347 (18.50%)	86 (17.40%)	93 (18.80%)
		Very low	402 (2.20%)	5 (1.00%)	4 (0.80%)
	Depression (n, %)	Yes	416 (2.30%)	11 (2.20%)	21 (4.20%)
		No	17,662 (97.70%)	484 (97.80%)	474 (95.80%)
Daejeon	Total		17,787	554	554
	Age (yrs, sd)		46.2 (15.8)	37.9 (15.4)	37.8 (15.8)
	Sex (n, %)	Male	8,178 (46.00%)	213 (38.40%)	225 (40.60%)
		Female	9,609 (54.00%)	341 (61.60%)	329 (59.40%)
	BMI (kg/m^2, sd)		22.8 (3.1)	22.4 (2.9)	22.3 (3.2)
	Education level (n, %)	No education	601 (3.60%)	8 (1.50%)	4 (0.80%)
		Pre-school	23 (0.10%)	0 (0.00%)	0 (0.00%)
		Elementary school	1,875 (11.20%)	25 (4.70%)	35 (6.70%)
		Middle school	1,713 (10.20%)	39 (1.20%)	34 (6.50%)

		High school	5,239 (31.30%)	128 (24.30%)	144 (27.40%)
		2-3yrs college	2,101 (12.60%)	80 (15.20%)	79 (15.00%)
		4yrs university	5,161 (30.90%)	247 (46.90%)	230 (43.70%)
	Current drinking (n, %)	Yes	12,649 (71.10%)	440 (79.40%)	438 (79.10%)
		No	5,138 (28.90%)	114 (20.60%)	116 (20.90%)
	Current smoking (n, %)	Yes	3,472 (19.50%)	80 (14.40%)	109 (19.70%)
		No	14,315 (80.50%)	474 (85.60%)	445 (80.30%)
	Sodium intake (n, %)	Very high	92 (0.50%)	5 (0.90%)	12 (2.20%)
		High	4,011 (22.60%)	150 (27.10%)	143 (25.80%)
		Moderate	9,460 (53.20%)	278 (50.20%)	264 (47.70%)
		Low	3,901 (21.90%)	112 (20.20%)	123 (22.20%)
		Very low	322 (1.80%)	9 (1.60%)	12 (2.20%)
	Depression (n, %)	Yes	417 (2.30%)	13 (2.30%)	29 (5.20%)
		No	17,370 (97.70%)	541 (97.70%)	525 (94.80%)
Ulsan	Total		17,919	392	392
	Age (yrs, sd)		46.3 (14.9)	37.6 (14.3)	37.6 (14.7)
	Sex (n, %)	Male	8,427 (47.00%)	160 (40.80%)	156 (39.80%)
		Female	9,492 (53.00%)	232 (59.20%)	236 (60.20%)
	BMI (kg/m^2, sd)		23 (3)	22.3 (2.9)	22.3 (3.2)
	Education level (n, %)	No education	685 (3.90%)	7 (1.80%)	2 (0.50%)
		Pre-school	27 (0.20%)	0 (0.00%)	1 (0.30%)
		Elementary school	1,791 (10.20%)	14 (3.70%)	28 (7.30%)
		Middle school	1,864 (10.60%)	17 (0.50%)	22 (5.70%)
		High school	6,994 (39.80%)	118 (30.90%)	118 (30.80%)
		2-3yrs college	2,528 (14.40%)	91 (23.80%)	88 (23.00%)
		4yrs university	3,693 (21.00%)	135 (35.30%)	124 (32.40%)
	Current drinking (n, %)	Yes	13,254 (74.00%)	326 (83.20%)	311 (79.30%)
		No	4,665 (26.00%)	66 (16.80%)	81 (20.70%)
	Current smoking (n, %)	Yes	3,832 (21.40%)	67 (17.10%)	79 (20.20%)
		No	14,087 (78.60%)	325 (82.90%)	313 (79.80%)
	Sodium intake (n, %)	Very high	103 (0.60%)	3 (0.80%)	4 (1.00%)
		High	4,353 (24.30%)	124 (31.60%)	118 (30.10%)

		Moderate	9,431 (52.60%)	188 (48.00%)	190 (48.50%)
		Low	3,690 (20.60%)	72 (18.40%)	75 (19.10%)
		Very low	342 (1.90%)	5 (1.30%)	5 (1.30%)
	Depression (n, %)	Yes	302 (1.70%)	386 (98.50%)	376 (95.90%)
		No	17,617 (98.30%)	6 (1.50%)	16 (4.10%)
Gyeonggido	Total		159,810	5,079	5,079
	Age (yrs, sd)		47.2 (16)	39.8 (16.9)	39.4 (16.7)
	Sex (n, %)	Male	73,254 (45.80%)	2,044 (40.20%)	2,013 (39.60%)
		Female	86,556 (54.20%)	3,035 (59.80%)	3,066 (60.40%)
	BMI (kg/m^2, sd)		23 (3.2)	22.5 (3.1)	22.6 (3.4)
	Education level (n, %)	No education	5,713 (3.70%)	104 (2.20%)	109 (2.30%)
		Pre-school	243 (0.20%)	2 (0.00%)	4 (0.10%)
		Elementary school	17,514 (11.50%)	382 (7.90%)	364 (7.60%)
		Middle school	14,980 (9.80%)	292 (9.20%)	286 (5.90%)
		High school	53,739 (35.20%)	1,367 (28.30%)	1,388 (28.80%)
		2-3yrs college	19,897 (13.00%)	875 (18.10%)	870 (18.10%)
		4yrs university	40,777 (26.70%)	1,814 (37.50%)	1,796 (37.30%)
	Current drinking (n, %)	Yes	114,433 (71.60%)	3,917 (77.10%)	3,914 (77.10%)
		No	45,377 (28.40%)	1,162 (22.90%)	1,165 (22.90%)
	Current smoking (n, %)	Yes	33,111 (20.70%)	859 (16.90%)	950 (18.70%)
		No	126,699 (79.30%)	4,220 (83.10%)	4,129 (81.30%)
	Sodium intake (n, %)	Very high	1,556 (1.00%)	65 (1.30%)	76 (1.50%)
		High	41,150 (25.80%)	1,561 (30.70%)	1,584 (31.20%)
		Moderate	79,280 (49.60%)	2,265 (44.60%)	2,206 (43.50%)
		Low	33,869 (21.20%)	1,091 (21.50%)	1,081 (21.30%)
		Very low	3,923 (2.50%)	97 (1.90%)	130 (2.60%)
	Depression (n, %)	Yes	3,884 (2.40%)	90 (1.80%)	259 (5.10%)
		No	155,926 (97.60%)	4,989 (98.20%)	4,820 (94.90%)
Gangwondo	Total		62,503	1,221	1,221
	Age (yrs, sd)		53.1 (16.3)	44.2 (17.9)	44.5 (18.3)
	Sex (n, %)	Male	29,180 (46.70%)	514 (42.10%)	499 (40.90%)
		Female	33,323 (53.30%)	707 (57.90%)	722 (59.10%)

	BMI (kg/m2, sd)		23.3 (3.4)	22.9 (3.1)	23.2 (3.8)
	Education level (n, %)	No education	5,385 (8.80%)	58 (4.80%)	66 (5.50%)
		Pre-school	194 (0.30%)	1 (0.10%)	6 (0.50%)
		Elementary school	14,438 (23.60%)	193 (16.10%)	182 (15.20%)
		Middle school	8,516 (13.90%)	128 (4.00%)	111 (9.30%)
		High school	18,210 (29.80%)	340 (28.30%)	342 (28.60%)
		2-3yrs college	5,441 (8.90%)	171 (14.20%)	176 (14.70%)
		4yrs university	8,973 (14.70%)	311 (25.90%)	314 (26.20%)
	Current drinking (n, %)	Yes	40,649 (65.00%)	882 (72.20%)	866 (70.90%)
		No	21,854 (35.00%)	339 (27.80%)	355 (29.10%)
	Current smoking (n, %)	Yes	13,175 (21.10%)	208 (17.00%)	231 (18.90%)
		No	49,328 (78.90%)	1,013 (83.00%)	990 (81.10%)
	Sodium intake (n, %)	Very high	703 (1.10%)	12 (1.00%)	21 (1.70%)
		High	16,283 (26.10%)	354 (29.00%)	362 (29.60%)
		Moderate	31,442 (50.30%)	590 (48.30%)	570 (46.70%)
		Low	12,700 (20.30%)	247 (20.20%)	232 (19.00%)
		Very low	1,366 (2.20%)	18 (1.50%)	36 (2.90%)
	Depression (n, %)	Yes	1,664 (2.70%)	28 (2.30%)	72 (5.90%)
		No	60,839 (97.30%)	1,193 (97.70%)	1,149 (94.10%)
Chungcheongbukdo	Total		45,631	908	908
	Age (yrs, sd)		52.9 (16.8)	44.3 (18.4)	44 (18.2)
	Sex (n, %)	Male	21,064 (46.20%)	352 (38.80%)	335 (36.90%)
		Female	24,567 (53.80%)	556 (61.20%)	573 (63.10%)
	BMI (kg/m2, sd)		22.9 (3.5)	22.7 (3.2)	22.9 (3.8)
	Education level (n, %)	No education	4,215 (9.50%)	43 (4.80%)	42 (4.80%)
		Pre-school	105 (0.20%)	0 (0.00%)	0 (0.00%)
		Elementary school	10,507 (23.60%)	157 (17.60%)	152 (17.30%)
		Middle school	5,796 (13.00%)	91 (2.90%)	93 (10.60%)
		High school	13,393 (30.10%)	229 (25.70%)	237 (27.00%)
		2-3yrs college	4,081 (9.20%)	134 (15.00%)	135 (15.40%)
		4yrs university	6,464 (14.50%)	238 (26.70%)	219 (24.90%)
	Current drinking (n, %)	Yes	29,383 (64.40%)	662 (72.90%)	637 (70.20%)
		No	16,248	246	271

			(35.60%)	(27.10%)	(29.80%)
	Current smoking (n, %)	Yes	9,659 (21.20%)	147 (16.20%)	162 (17.80%)
		No	35,972 (78.80%)	761 (83.80%)	746 (82.20%)
	Sodium intake (n, %)	Very high	595 (1.30%)	19 (2.10%)	17 (1.90%)
		High	12,920 (28.30%)	270 (29.80%)	289 (31.80%)
		Moderate	21,875 (47.90%)	421 (46.40%)	397 (43.70%)
		Low	9,273 (20.30%)	177 (19.50%)	190 (20.90%)
		Very low	958 (2.10%)	20 (2.20%)	15 (1.70%)
	Depression (n, %)	Yes	1,134 (2.50%)	26 (2.90%)	53 (5.80%)
		No	44,497 (97.50%)	882 (97.10%)	855 (94.20%)
Chungcheongnamdo	Total		54,682	1,165	1,165
	Age (yrs, sd)		53.8 (17.1)	46.4 (18.6)	46.3 (18.6)
	Sex (n, %)	Male	24,879 (45.50%)	491 (42.10%)	486 (41.70%)
		Female	29,803 (54.50%)	674 (57.90%)	679 (58.30%)
	BMI (kg/m^2, sd)		22.9 (3.3)	22.8 (3.1)	22.9 (3.5)
	Education level (n, %)	No education	6,121 (11.50%)	90 (7.90%)	79 (7.00%)
		Pre-school	173 (0.30%)	0 (0.00%)	3 (0.30%)
		Elementary school	13,613 (25.50%)	225 (19.60%)	233 (20.50%)
		Middle school	6,418 (12.00%)	93 (2.90%)	100 (8.80%)
		High school	14,917 (28.00%)	304 (26.50%)	303 (26.70%)
		2-3yrs college	4,730 (8.90%)	149 (13.00%)	150 (13.20%)
		4yrs university	7,365 (13.80%)	285 (24.90%)	267 (23.50%)
	Current drinking (n, %)	Yes	32,093 (58.70%)	754 (64.70%)	740 (63.50%)
		No	22,589 (41.30%)	411 (35.30%)	425 (36.50%)
	Current smoking (n, %)	Yes	10,330 (18.90%)	217 (18.60%)	230 (19.70%)
		No	44,352 (81.10%)	948 (81.40%)	935 (80.30%)
	Sodium intake (n, %)	Very high	543 (1.00%)	13 (1.10%)	20 (1.70%)
		High	13,778 (25.20%)	344 (29.50%)	341 (29.30%)
		Moderate	28,254 (51.70%)	541 (46.40%)	520 (44.60%)
		Low	11,120 (20.30%)	250 (21.50%)	255 (21.90%)
		Very low	981 (1.80%)	17 (1.50%)	29 (2.50%)
	Depression (n, %)	Yes	1,426 (2.60%)	25 (2.10%)	52

					(4.50%)
		No	53,256 (97.40%)	1,140 (97.90%)	1,113 (95.50%)
Jeollabukdo	Total		49,498	734	734
	Age (yrs, sd)		56.5 (16.9)	48.6 (19.3)	47.9 (18.7)
	Sex (n, %)	Male	21,976 (44.40%)	295 (40.20%)	313 (42.60%)
		Female	27,522 (55.60%)	439 (59.80%)	421 (57.40%)
	BMI (kg/m^2, sd)		22.4 (4)	22.5 (3.2)	22.8 (3.8)
	Education level (n, %)	No education	8,252 (17.00%)	77 (10.60%)	68 (9.60%)
		Pre-school	221 (0.50%)	1 (0.10%)	1 (0.10%)
		Elementary school	13,116 (27.00%)	165 (22.80%)	148 (20.90%)
		Middle school	5,955 (12.20%)	66 (2.10%)	73 (10.30%)
		High school	11,737 (24.10%)	174 (24.00%)	177 (25.00%)
		2-3yrs college	3,226 (6.60%)	64 (8.80%)	72 (10.20%)
		4yrs university	6,158 (12.70%)	178 (24.60%)	168 (23.80%)
	Current drinking (n, %)	Yes	26,798 (54.10%)	459 (62.50%)	456 (62.10%)
		No	22,700 (45.90%)	275 (37.50%)	278 (37.90%)
	Current smoking (n, %)	Yes	8,685 (17.50%)	128 (17.40%)	149 (20.30%)
		No	40,813 (82.50%)	606 (82.60%)	585 (79.70%)
	Sodium intake (n, %)	Very high	386 (0.80%)	8 (1.10%)	9 (1.20%)
		High	12,432 (25.10%)	200 (27.20%)	210 (28.70%)
		Moderate	26,595 (53.80%)	376 (51.20%)	360 (49.20%)
		Low	9,317 (18.80%)	144 (19.60%)	139 (19.00%)
		Very low	738 (1.50%)	6 (0.80%)	14 (1.90%)
	Depression (n, %)	Yes	1,235 (2.50%)	10 (1.40%)	40 (5.40%)
		No	48,263 (97.50%)	724 (98.60%)	694 (94.60%)
Jeollanamdo	Total		78,491	1,124	1,124
	Age (yrs, sd)		57.3 (16.7)	49.5 (19.5)	49.2 (19.5)
	Sex (n, %)	Male	34,645 (44.10%)	474 (42.20%)	470 (41.80%)
		Female	43,846 (55.90%)	650 (57.80%)	654 (58.20%)
	BMI (kg/m^2, sd)		22.6 (3.7)	22.6 (3.2)	22.7 (3.6)
	Education level (n, %)	No education	13,239 (17.10%)	114 (10.30%)	112 (10.20%)
		Pre-school	486 (0.60%)	3 (0.30%)	6 (0.50%)
		Elementary school	21,842 (28.20%)	261 (23.50%)	242 (22.10%)
		Middle school	10,291	118 (3.70%)	118

			(13.30%)		(10.80%)
		High school	18,134 (23.40%)	267 (24.10%)	261 (23.80%)
		2-3yrs college	5,706 (7.40%)	144 (13.00%)	156 (14.20%)
		4yrs university	7,733 (10.00%)	203 (18.30%)	202 (18.40%)
	Current drinking (n, %)	Yes	40,677 (51.80%)	658 (58.50%)	683 (60.80%)
		No	37,814 (48.20%)	466 (41.50%)	441 (39.20%)
	Current smoking (n, %)	Yes	13,134 (16.70%)	179 (15.90%)	194 (17.30%)
		No	65,357 (83.30%)	945 (84.10%)	930 (82.70%)
	Sodium intake (n, %)	Very high	484 (0.60%)	13 (1.20%)	19 (1.70%)
		High	16,130 (20.60%)	292 (26.00%)	291 (25.90%)
		Moderate	45,553 (58.10%)	594 (52.80%)	584 (52.00%)
		Low	15,033 (19.20%)	209 (18.60%)	205 (18.30%)
		Very low	1,266 (1.60%)	16 (1.40%)	23 (2.00%)
	Depression (n, %)	Yes	1,729 (2.20%)	28 (2.50%)	54 (4.80%)
		No	76,762 (97.80%)	1,096 (97.50%)	1,070 (95.20%)
Gyeongsangbukdo	Total		87,813	1,381	1,381
	Age (yrs, sd)		55.4 (16.5)	47.1 (18.6)	46.7 (18.3)
	Sex (n, %)	Male	39,614 (45.10%)	558 (40.40%)	552 (40.00%)
		Female	48,199 (54.90%)	823 (59.60%)	829 (60.00%)
	BMI (kg/m^2, sd)		22.8 (3.4)	22.5 (3.1)	22.6 (3.2)
	Education level (n, %)	No education	10,147 (11.70%)	106 (7.70%)	87 (6.40%)
		Pre-school	264 (0.30%)	2 (0.10%)	2 (0.10%)
		Elementary school	23,059 (26.70%)	248 (18.10%)	250 (18.50%)
		Middle school	12,097 (14.00%)	156 (4.90%)	168 (12.40%)
		High school	22,910 (26.50%)	358 (26.20%)	345 (25.50%)
		2-3yrs college	8,304 (9.60%)	204 (14.90%)	206 (15.20%)
		4yrs university	9,649 (11.20%)	294 (21.50%)	296 (21.90%)
	Current drinking (n, %)	Yes	53,252 (60.60%)	909 (65.80%)	902 (65.30%)
		No	34,561 (39.40%)	472 (34.20%)	479 (34.70%)
	Current smoking (n, %)	Yes	17,655 (20.10%)	253 (18.30%)	280 (20.30%)
		No	70,158 (79.90%)	1,128 (81.70%)	1,101 (79.70%)
	Sodium intake (n, %)	Very high	884 (1.00%)	16 (1.20%)	23

					(1.70%)
		High	23,635 (26.90%)	420 (30.40%)	387 (28.00%)
		Moderate	42,559 (48.50%)	623 (45.10%)	629 (45.50%)
		Low	18,738 (21.30%)	302 (21.90%)	304 (22.00%)
		Very low	1,982 (2.30%)	20 (1.40%)	38 (2.80%)
	Depression (n, %)	Yes	2,145 (2.40%)	35 (2.50%)	73 (5.30%)
		No	85,668 (97.60%)	1,346 (97.50%)	1,308 (94.70%)
Gyeongsangnamdo	Total		71,225	1,209	1,209
	Age (yrs, sd)		53.9 (16.9)	45.6 (18.7)	44.8 (18.2)
	Sex (n, %)	Male	31,487 (44.20%)	490 (40.50%)	498 (41.20%)
		Female	39,738 (55.80%)	719 (59.50%)	711 (58.80%)
	BMI (kg/m^2, sd)		22.6 (3.6)	22.4 (3.1)	22.6 (3.4)
	Education level (n, %)	No education	8,921 (12.80%)	90 (7.50%)	63 (5.40%)
		Pre-school	219 (0.30%)	4 (0.30%)	4 (0.30%)
		Elementary school	15,117 (21.60%)	167 (14.00%)	165 (14.10%)
		Middle school	8,777 (12.60%)	105 (3.30%)	107 (9.10%)
		High school	20,476 (29.30%)	339 (28.30%)	341 (29.00%)
		2-3yrs college	6,475 (9.30%)	181 (15.10%)	189 (16.10%)
		4yrs university	9,932 (14.20%)	310 (25.90%)	305 (26.00%)
	Current drinking (n, %)	Yes	44,727 (62.80%)	840 (69.50%)	850 (70.30%)
		No	26,498 (37.20%)	369 (30.50%)	359 (29.70%)
	Current smoking (n, %)	Yes	13,954 (19.60%)	208 (17.20%)	214 (17.70%)
		No	57,271 (80.40%)	1001 (82.80%)	995 (82.30%)
	Sodium intake (n, %)	Very high	517 (0.70%)	7 (0.60%)	10 (0.80%)
		High	19,089 (26.80%)	403 (33.30%)	375 (31.00%)
		Moderate	35,724 (50.20%)	535 (44.30%)	548 (45.30%)
		Low	14,561 (20.40%)	255 (21.10%)	252 (20.80%)
		Very low	1,328 (1.90%)	9 (0.70%)	24 (2.00%)
	Depression (n, %)	Yes	1,522 (2.10%)	15 (1.20%)	63 (5.20%)
		No	69,703 (97.90%)	1,194 (98.80%)	1,146 (94.80%)
Jejudo	Total		19,952	459	459
	Age (yrs, sd)		53.6 (16.6)	46.4 (19.6)	46.3 (20.1)

Sex (n, %)	Male	9,177 (46.00%)	196 (42.70%)	196 (42.70%)
	Female	10,775 (54.00%)	263 (57.30%)	263 (57.30%)
BMI (kg/m^2, sd)		23.3 (3.4)	22.9 (3.3)	22.7 (3.5)
Education level (n, %)	No education	2,129 (10.90%)	32 (7.20%)	27 (6.00%)
	Pre-school	205 (1.00%)	1 (0.20%)	4 (0.90%)
	Elementary school	3,561 (18.20%)	58 (13.00%)	66 (14.70%)
	Middle school	2,463 (12.60%)	56 (1.80%)	60 (13.40%)
	High school	5,936 (30.40%)	97 (21.70%)	88 (19.60%)
	2-3yrs college	2,652 (13.60%)	109 (24.40%)	109 (24.30%)
	4yrs university	2,585 (13.20%)	93 (20.90%)	94 (21.00%)
Current drinking (n, %)	Yes	11,582 (58.00%)	301 (65.60%)	287 (62.50%)
	No	8,370 (42.00%)	158 (34.40%)	172 (37.50%)
Current smoking (n, %)	Yes	4,163 (20.90%)	86 (18.70%)	87 (19.00%)
	No	15,789 (79.10%)	373 (81.30%)	372 (81.00%)
Sodium intake (n, %)	Very high	132 (0.70%)	4 (0.90%)	5 (1.10%)
	High	4,349 (21.80%)	121 (26.40%)	125 (27.20%)
	Moderate	10,238 (51.30%)	221 (48.30%)	223 (48.60%)
	Low	4,702 (23.60%)	106 (23.10%)	98 (21.40%)
	Very low	519 (2.60%)	6 (1.30%)	8 (1.70%)
Depression (n, %)	Yes	559 (2.80%)	11 (2.40%)	23 (5.00%)
	No	19,393 (97.20%)	448 (97.60%)	436 (95.00%)

Table S2. Prevalence of atopic dermatitis (AD) and depression diagnosis in 253 study communities in South Korea

		Prevalence (%)	
**Region**		**AD**	**Depression**
Seoul	Gangnam-gu	2.72	2.21
	Gangdong-gu	3.90	2.33
	Gangbuk-gu	3.83	2.99
	Gangseo-gu	3.38	2.62
	Gwanak-gu	4.58	2.86
	Gwangjin-gu	3.55	2.28
	Guro-gu	2.79	2.73
	Geumcheon-gu	2.88	2.29
	Nowon-gu	2.98	3.22
	Dobong-gu	3.46	2.49
	Dongdaemun-gu	3.52	2.82
	Dongjak-gu	3.97	2.80
	Mapo-gu	4.74	2.43
	Seodaemun-gu	4.58	1.92
	Seocho-gu	4.36	2.63
	Seongdong-gu	3.52	2.66
	Seongbuk-gu	4.24	3.95
	Songpa-gu	4.51	2.19
	Yangcheon-gu	3.57	2.29
	Yeongdeungpo-gu	4.41	2.48
	Yongsan-gu	3.07	2.50
	Eunpyeong-gu	3.62	2.24
	Jongno-gu	4.27	3.23
	Jung-gu	2.96	1.71
	Jungnang-gu	4.01	3.60
Busan	Gangseo-gu	1.73	3.23
	Gumjung-gu	2.15	1.63
	Gijang-gun	2.77	2.77
	Nam-gu	1.93	2.31
	Dong-gu	1.76	3.03
	Dongnae-gu	1.85	2.67
	Busanjin-gu	2.36	2.80
	Buk-gu	1.69	1.72
	Sasang-gu	1.91	2.75
	Saha-gu	2.45	1.77
	Seo-gu	1.81	2.36
	Suyeong-gu	2.22	2.71
	Yeonje-gu	3.03	3.47
	Yeongdo-gu	2.11	3.47
	Jung-gu	2.33	2.02
	Haeundae-gu	3.08	2.65
Daegu	Nam-gu	2.27	3.25
	Dalseo-gu	2.53	2.91
	Dalseong-gun	2.11	2.27
	Dong-gu	3.02	3.24
	Buk-gu	2.42	2.75
	Seo-gu	1.36	2.43
	Suseong-gu	2.56	2.07
	Jung-gu	2.95	3.58
Incheon	Ganghwa-gun	3.20	2.79
	Gyeyang-gu	2.93	2.25
	Nam-gu	3.88	2.60
	Namdong-gu	1.34	1.43
	Dong-gu	1.94	2.36

	Bupyeong-gu	2.97	1.84
	Seo-gu	2.16	2.45
	Yeonsu-gu	2.71	2.85
	Ongjin-gun	2.82	2.44
	Jung-gu	2.68	2.19
Gwangju	Gwangsan-gu	1.69	1.99
	Nam-gu	3.44	3.22
	Dong-gu	2.08	2.30
	Buk-gu	2.88	2.80
	Seo-gu	2.13	1.99
Daejeon	Daedeok-gu	2.56	1.86
	Dong-gu	2.45	2.34
	Yuseong-gu	3.48	2.61
	Seo-gu	3.11	1.58
	Jung-gu	3.49	3.77
Ulsan	Nam-gu	2.48	1.99
	Dong-gu	2.13	1.39
	Buk-gu	2.41	1.23
	Ulju-gun	1.83	1.80
	Jung-gu	1.86	2.27
Gyeonggi-do	Gapyeong-gun	2.03	3.26
	Goyang-si Deogyang-gu	3.16	2.51
	Goyang-si Ilsan-dong-gu	3.40	2.85
	Goyang-si Ilsan-seo-gu	4.51	3.01
	Gwacheon-si	3.50	2.09
	Guri-si	3.10	2.52
	Gimpo-si	3.26	1.89
	Namyangju-si	3.19	2.78
	Dongducheon-si	2.30	2.49
	Bucheon-si Sosa-gu	3.12	1.83
	Bucheon-si Ojeong-gu	2.44	1.53
	Bucheon-si Wonmi-gu	3.39	3.17
	Yangju-gun	2.62	2.93
	Yangpyeong-gun	2.89	2.81
	Yeoju-gun	2.89	2.65
	Yeoncheon-gun	1.87	3.04
	Uijeongbu-si	3.11	2.87
	Icheon-si	2.19	2.55
	Paju-si	2.46	2.13
	Pocheon-gun	2.32	3.26
	Hanam-si	3.35	2.80
	Gwangmyeong-si	3.26	2.17
	Gwangju-gun	2.51	1.66
	Gunpo-si	4.00	2.91
	Seongnam-si Bundang-gu	3.37	2.53
	Seongnam-si Sujeong-gu	2.70	2.81
	Seongnam-si Jungwon-gu	3.12	2.28
	Suwon-si Gwonseon-gu	3.43	2.38
	Suwon-si Youngtong-gu	3.82	2.35
	Suwon-si Jangan-gu	3.23	2.06
	Suwon-si Paldal-gu	3.36	2.46
	Siheung-si	2.47	1.88
	Ansan-si Danwon	3.69	2.74
	Ansan-si Sangroksu	3.96	3.12
	Anseong-si	3.01	3.47
	Anyang-si Dongan-gu	3.43	2.38
	Anyang-si Manan-gu	3.70	2.94
	Osan-si	2.59	1.75
	Yongin-si Giheung-gu	3.98	2.77

	Yongin-si Suji-gu	2.95	2.60
	Yongin-si Cheoin-gu	2.45	2.10
	Uiwang-si	4.19	2.23
	Pyeongtaek-si Songtan	2.71	2.20
	Pyeongtaek-si Pyeongtaek	2.73	2.60
	Hwaseong-si	2.74	1.76
Gangwon-do	Gangneung-si	2.33	2.08
	Goseong-gun	2.59	2.51
	Donghae-si	2.06	3.23
	Samcheok-si	1.93	3.01
	Sokcho-si	2.66	3.27
	Yanggu-gun	1.72	2.13
	Yangyang-gun	1.40	3.58
	Yeongwol-gun	1.85	3.30
	Wonju-si	2.55	2.68
	Inje-gun	1.35	1.98
	Jeongseon-gun	1.36	2.60
	Cheorwon-gun	1.77	2.31
	Chuncheon-si	1.77	2.41
	Taebaek-si	1.92	2.69
	Pyeongchang-gun	1.47	2.32
	Hongcheon-gun	1.91	3.34
	Hwacheon-gun	1.77	2.44
	Hoengseong-gun	2.03	3.13
Chungcheongbuk-do	Goesan-gun	1.11	2.32
	Danyang-gun	0.97	1.80
	Boeun-gun	1.51	2.79
	Yeongdong-gun	1.42	2.45
	Okcheon-gun	3.25	4.20
	Eumseong-gun	2.50	2.19
	Jecheon-si	1.55	2.16
	Jeungpyong-gun	2.29	2.43
	Jincheon-gun	1.48	2.38
	Cheongwon-gun	2.32	2.18
	Cheongju-si Sangdang	2.63	2.60
	Cheongju-si Heungdeok	2.20	2.39
	Chungju-si	2.07	3.23
Chungcheongnam-do	Gyeryong-si	3.28	1.97
	Gongju-si	1.85	3.20
	Geumsan-gun	2.29	2.52
	Nonsan-si	2.03	3.35
	Dangjin-gun	2.02	2.46
	Boryeong-si	1.91	2.83
	Buyeo-gun	1.61	1.91
	Seosan-si	1.24	1.43
	Seocheon-gun	1.59	3.78
	Asan-si	2.91	2.94
	Yeongi-gun	2.11	3.67
	Yesan-gun	2.11	2.69
	Cheonan-si	2.44	2.27
	Cheongyang-gun	1.66	2.48
	Taean-gun	1.79	2.21
	Hongseong-gun	2.58	3.13
Jeollabuk-do	Gochang-gun	1.42	3.50
	Gunsan-si	1.87	1.79
	Gimje-si	1.99	2.86
	Namwon-si	1.69	2.39
	Muju-gun	1.35	2.85
	Buan-gun	1.32	3.30

	Sunchang-gun	1.45	2.95
	Wanju-gun	1.13	1.89
	Iksan-si	2.32	2.29
	Imsil-gun	0.94	2.41
	Jangsu-gun	1.01	2.40
	Jeonju-si	2.16	2.02
	Jeongeup-si	1.32	2.68
	Jinan-gun	0.40	2.25
Jeollanam-do	Gangjin-gun	0.86	1.24
	Goheung-gun	0.68	1.72
	Gokseong-gun	2.11	3.78
	Gwangyang-si	1.90	1.35
	Gurye-gun	0.74	1.79
	Naju-si	1.63	2.41
	Damyang-gun	1.44	3.23
	Mokpo-si	2.17	2.28
	Muan-gun	1.41	1.47
	Boseong-gun	1.28	2.44
	Suncheon-si	1.34	1.48
	Sinan-gun	0.67	1.96
	Yeosu-si	1.97	1.70
	Yeonggwang-gun	1.44	4.45
	Yeongam-gun	1.23	2.24
	Wando-gun	1.48	2.09
	Jangseong-gun	1.51	2.41
	Jangheung-gun	0.67	1.14
	Jindo-gun	0.47	1.84
	Hampyeong-gun	1.68	3.18
	Haenam-gun	1.62	2.36
	Hwasun-gun	2.73	2.76
Gyeongsangbuk-do	Gyeongsan-si	2.20	2.69
	Gyeongju-si	1.35	2.62
	Goryeong-gun	1.45	3.44
	Gumi-si gumi	3.20	2.09
	Gumi-si sunsan	1.55	2.25
	Gunwi-gun	0.97	2.40
	Gimcheon-si	1.36	2.68
	Mungyeong-si	1.59	2.45
	Bonghwa-gun	1.05	2.49
	Sangju-si	2.14	2.77
	Seongju-gun	0.70	1.59
	Andong-si	3.06	3.42
	Yeongdeok-gun	0.84	1.80
	Yeongyang-gun	1.19	2.07
	Yeongju-si	1.97	2.63
	Yeongcheon-si	1.63	3.90
	Yecheon-gun	0.92	2.04
	Ulleung-gun	0.78	1.49
	Uljin-gun	1.29	1.90
	Uiseong-gun	1.25	2.54
	Cheongdo-gun	1.35	3.40
	Cheongsong-gun	1.60	2.88
	Chilgok-gun	1.83	2.75
	Pohang-si nam-gu	1.84	1.81
	Pohang-si buk-gu	1.43	1.95
Gyeongsangnam-do	Geoje-si	1.64	1.67
	Geochang-gun	1.38	2.51
	Goseong-gun	1.46	2.36
	Gimhae-si	1.82	1.56

	Namhae-gun	1.15	3.20
	Masan-si	2.55	1.96
	Miryang-si	1.28	1.77
	Sacheon-si	1.47	2.61
	Sancheong-gun	0.65	1.75
	Yangsan-si	1.91	1.77
	Uiryeong-gun	0.88	1.99
	Jinju-si	2.58	2.88
	Jinhae-si	2.04	1.79
	Changnyeong-gun	0.71	2.63
	Changwon-si	2.87	1.66
	Tongyeong-si	1.55	2.02
	Hadong-gun	2.46	3.22
	Haman-gun	3.17	2.67
	Hamyang-gun	0.98	1.90
	Hapcheon-gun	0.70	1.90
Jeju-do	Seogwipo-si Seogwipo	1.96	3.21
	Seogwipo-si dongbu	1.74	2.15
	Seogwipo-siseobu	2.02	3.19
	Jeju-si jeju	3.21	2.99
	Jeju-si dongbu	1.65	2.97
	Jeju-si seobu	2.87	2.61
