# Supplementary material for: Estimating causal associations of atopic dermatitis with depression using the propensity score method: an analysis of Korea Community Health Survey data, 2010-2013
Source: Epidemiol Health. 2018 Nov 29;40:e2018059. doi: 10.4178/epih.e2018059 (PMC6335500; doi:10.4178/epih.e2018059)
Supplement: Supplementary file 2 [file epih-40-e2018059-supplementary2.pdf]

Supplementary Material 2

Table S2. Prevalence of atopic dermatitis (AD) and depression diagnosis in 253 study communities in South Korea

		Prevalence (%)	
**Region**		**AD**	**Depression**
Seoul	Gangnam-gu	2.72	2.21
	Gangdong-gu	3.90	2.33
	Gangbuk-gu	3.83	2.99
	Gangseo-gu	3.38	2.62
	Gwanak-gu	4.58	2.86
	Gwangjin-gu	3.55	2.28
	Guro-gu	2.79	2.73
	Geumcheon-gu	2.88	2.29
	Nowon-gu	2.98	3.22
	Dobong-gu	3.46	2.49
	Dongdaemun-gu	3.52	2.82
	Dongjak-gu	3.97	2.80
	Mapo-gu	4.74	2.43
	Seodaemun-gu	4.58	1.92
	Seocho-gu	4.36	2.63
	Seongdong-gu	3.52	2.66
	Seongbuk-gu	4.24	3.95
	Songpa-gu	4.51	2.19
	Yangcheon-gu	3.57	2.29
	Yeongdeungpo-gu	4.41	2.48
	Yongsan-gu	3.07	2.50
	Eunpyeong-gu	3.62	2.24
	Jongno-gu	4.27	3.23
	Jung-gu	2.96	1.71
	Jungnang-gu	4.01	3.60
Busan	Gangseo-gu	1.73	3.23
	Gumjung-gu	2.15	1.63
	Gijang-gun	2.77	2.77
	Nam-gu	1.93	2.31
	Dong-gu	1.76	3.03
	Dongnae-gu	1.85	2.67
	Busanjin-gu	2.36	2.80
	Buk-gu	1.69	1.72
	Sasang-gu	1.91	2.75
	Saha-gu	2.45	1.77
	Seo-gu	1.81	2.36
	Suyeong-gu	2.22	2.71
	Yeonje-gu	3.03	3.47
	Yeongdo-gu	2.11	3.47
	Jung-gu	2.33	2.02
	Haeundae-gu	3.08	2.65
Daegu	Nam-gu	2.27	3.25
	Dalseo-gu	2.53	2.91
	Dalseong-gun	2.11	2.27
	Dong-gu	3.02	3.24
	Buk-gu	2.42	2.75
	Seo-gu	1.36	2.43
	Suseong-gu	2.56	2.07
	Jung-gu	2.95	3.58
Incheon	Ganghwa-gun	3.20	2.79

	Gyeyang-gu	2.93	2.25
	Nam-gu	3.88	2.60
	Namdong-gu	1.34	1.43
	Dong-gu	1.94	2.36
	Bupyeong-gu	2.97	1.84
	Seo-gu	2.16	2.45
	Yeonsu-gu	2.71	2.85
	Ongjin-gun	2.82	2.44
	Jung-gu	2.68	2.19
Gwangju	Gwangsan-gu	1.69	1.99
	Nam-gu	3.44	3.22
	Dong-gu	2.08	2.30
	Buk-gu	2.88	2.80
	Seo-gu	2.13	1.99
Daejeon	Daedeok-gu	2.56	1.86
	Dong-gu	2.45	2.34
	Yuseong-gu	3.48	2.61
	Seo-gu	3.11	1.58
	Jung-gu	3.49	3.77
Ulsan	Nam-gu	2.48	1.99
	Dong-gu	2.13	1.39
	Buk-gu	2.41	1.23
	Ulju-gun	1.83	1.80
	Jung-gu	1.86	2.27
Gyeonggi-do	Gapyeong-gun	2.03	3.26
	Goyang-si Deogyang-gu	3.16	2.51
	Goyang-si Ilsan-dong-gu	3.40	2.85
	Goyang-si Ilsan-seo-gu	4.51	3.01
	Gwacheon-si	3.50	2.09
	Guri-si	3.10	2.52
	Gimpo-si	3.26	1.89
	Namyangju-si	3.19	2.78
	Dongducheon-si	2.30	2.49
	Bucheon-si Sosa-gu	3.12	1.83
	Bucheon-si Ojeong-gu	2.44	1.53
	Bucheon-si Wonmi-gu	3.39	3.17
	Yangju-gun	2.62	2.93
	Yangpyeong-gun	2.89	2.81
	Yeoju-gun	2.89	2.65
	Yeoncheon-gun	1.87	3.04
	Uijeongbu-si	3.11	2.87
	Icheon-si	2.19	2.55
	Paju-si	2.46	2.13
	Pocheon-gun	2.32	3.26
	Hanam-si	3.35	2.80
	Gwangmyeong-si	3.26	2.17
	Gwangju-gun	2.51	1.66
	Gunpo-si	4.00	2.91
	Seongnam-si Bundang-gu	3.37	2.53
	Seongnam-si Sujeong-gu	2.70	2.81
	Seongnam-si Jungwon-gu	3.12	2.28
	Suwon-si Gwonseon-gu	3.43	2.38
	Suwon-si Youngtong-gu	3.82	2.35
	Suwon-si Jangan-gu	3.23	2.06
	Suwon-si Paldal-gu	3.36	2.46
	Siheung-si	2.47	1.88
	Ansan-si Danwon	3.69	2.74
	Ansan-si Sangroksu	3.96	3.12
	Anseong-si	3.01	3.47

	Anyang-si Dongan-gu	3.43	2.38
	Anyang-si Manan-gu	3.70	2.94
	Osan-si	2.59	1.75
	Yongin-si Giheung-gu	3.98	2.77
	Yongin-si Suji-gu	2.95	2.60
	Yongin-si Cheoin-gu	2.45	2.10
	Uiwang-si	4.19	2.23
	Pyeongtaek-si Songtan	2.71	2.20
	Pyeongtaek-si Pyeongtaek	2.73	2.60
	Hwaseong-si	2.74	1.76
Gangwon-do	Gangneung-si	2.33	2.08
	Goseong-gun	2.59	2.51
	Donghae-si	2.06	3.23
	Samcheok-si	1.93	3.01
	Sokcho-si	2.66	3.27
	Yanggu-gun	1.72	2.13
	Yangyang-gun	1.40	3.58
	Yeongwol-gun	1.85	3.30
	Wonju-si	2.55	2.68
	Inje-gun	1.35	1.98
	Jeongseon-gun	1.36	2.60
	Cheorwon-gun	1.77	2.31
	Chuncheon-si	1.77	2.41
	Taebaek-si	1.92	2.69
	Pyeongchang-gun	1.47	2.32
	Hongcheon-gun	1.91	3.34
	Hwacheon-gun	1.77	2.44
	Hoengseong-gun	2.03	3.13
Chungcheongbuk-do	Goesan-gun	1.11	2.32
	Danyang-gun	0.97	1.80
	Boeun-gun	1.51	2.79
	Yeongdong-gun	1.42	2.45
	Okcheon-gun	3.25	4.20
	Eumseong-gun	2.50	2.19
	Jecheon-si	1.55	2.16
	Jeungpyong-gun	2.29	2.43
	Jincheon-gun	1.48	2.38
	Cheongwon-gun	2.32	2.18
	Cheongju-si Sangdang	2.63	2.60
	Cheongju-si Heungdeok	2.20	2.39
	Chungju-si	2.07	3.23
Chungcheongnam-do	Gyeryong-si	3.28	1.97
	Gongju-si	1.85	3.20
	Geumsan-gun	2.29	2.52
	Nonsan-si	2.03	3.35
	Dangjin-gun	2.02	2.46
	Boryeong-si	1.91	2.83
	Buyeo-gun	1.61	1.91
	Seosan-si	1.24	1.43
	Seocheon-gun	1.59	3.78
	Asan-si	2.91	2.94
	Yeongi-gun	2.11	3.67
	Yesan-gun	2.11	2.69
	Cheonan-si	2.44	2.27
	Cheongyang-gun	1.66	2.48
	Taean-gun	1.79	2.21
	Hongseong-gun	2.58	3.13
Jeollabuk-do	Gochang-gun	1.42	3.50
	Gunsan-si	1.87	1.79

	Gimje-si	1.99	2.86
	Namwon-si	1.69	2.39
	Muju-gun	1.35	2.85
	Buan-gun	1.32	3.30
	Sunchang-gun	1.45	2.95
	Wanju-gun	1.13	1.89
	Iksan-si	2.32	2.29
	Imsil-gun	0.94	2.41
	Jangsu-gun	1.01	2.40
	Jeonju-si	2.16	2.02
	Jeongeup-si	1.32	2.68
	Jinan-gun	0.40	2.25
Jeollanam-do	Gangjin-gun	0.86	1.24
	Goheung-gun	0.68	1.72
	Gokseong-gun	2.11	3.78
	Gwangyang-si	1.90	1.35
	Gurye-gun	0.74	1.79
	Naju-si	1.63	2.41
	Damyang-gun	1.44	3.23
	Mokpo-si	2.17	2.28
	Muan-gun	1.41	1.47
	Boseong-gun	1.28	2.44
	Suncheon-si	1.34	1.48
	Sinan-gun	0.67	1.96
	Yeosu-si	1.97	1.70
	Yeonggwang-gun	1.44	4.45
	Yeongam-gun	1.23	2.24
	Wando-gun	1.48	2.09
	Jangseong-gun	1.51	2.41
	Jangheung-gun	0.67	1.14
	Jindo-gun	0.47	1.84
	Hampyeong-gun	1.68	3.18
	Haenam-gun	1.62	2.36
	Hwasun-gun	2.73	2.76
Gyeongsangbuk-do	Gyeongsan-si	2.20	2.69
	Gyeongju-si	1.35	2.62
	Goryeong-gun	1.45	3.44
	Gumi-si gumi	3.20	2.09
	Gumi-si sunsan	1.55	2.25
	Gunwi-gun	0.97	2.40
	Gimcheon-si	1.36	2.68
	Mungyeong-si	1.59	2.45
	Bonghwa-gun	1.05	2.49
	Sangju-si	2.14	2.77
	Seongju-gun	0.70	1.59
	Andong-si	3.06	3.42
	Yeongdeok-gun	0.84	1.80
	Yeongyang-gun	1.19	2.07
	Yeongju-si	1.97	2.63
	Yeongcheon-si	1.63	3.90
	Yecheon-gun	0.92	2.04
	Ulleung-gun	0.78	1.49
	Uljin-gun	1.29	1.90
	Uiseong-gun	1.25	2.54
	Cheongdo-gun	1.35	3.40
	Cheongsong-gun	1.60	2.88
	Chilgok-gun	1.83	2.75
	Pohang-si nam-gu	1.84	1.81
	Pohang-si buk-gu	1.43	1.95

Gyeongsangnam-do	Geoje-si	1.64	1.67
	Geochang-gun	1.38	2.51
	Goseong-gun	1.46	2.36
	Gimhae-si	1.82	1.56
	Namhae-gun	1.15	3.20
	Masan-si	2.55	1.96
	Miryang-si	1.28	1.77
	Sacheon-si	1.47	2.61
	Sancheong-gun	0.65	1.75
	Yangsan-si	1.91	1.77
	Uiryeong-gun	0.88	1.99
	Jinju-si	2.58	2.88
	Jinhae-si	2.04	1.79
	Changnyeong-gun	0.71	2.63
	Changwon-si	2.87	1.66
	Tongyeong-si	1.55	2.02
	Hadong-gun	2.46	3.22
	Haman-gun	3.17	2.67
	Hamyang-gun	0.98	1.90
	Hapcheon-gun	0.70	1.90
Jeju-do	Seogwipo-si Seogwipo	1.96	3.21
	Seogwipo-si dongbu	1.74	2.15
	Seogwipo-siseobu	2.02	3.19
	Jeju-si jeju	3.21	2.99
	Jeju-si dongbu	1.65	2.97
	Jeju-si seobu	2.87	2.61
